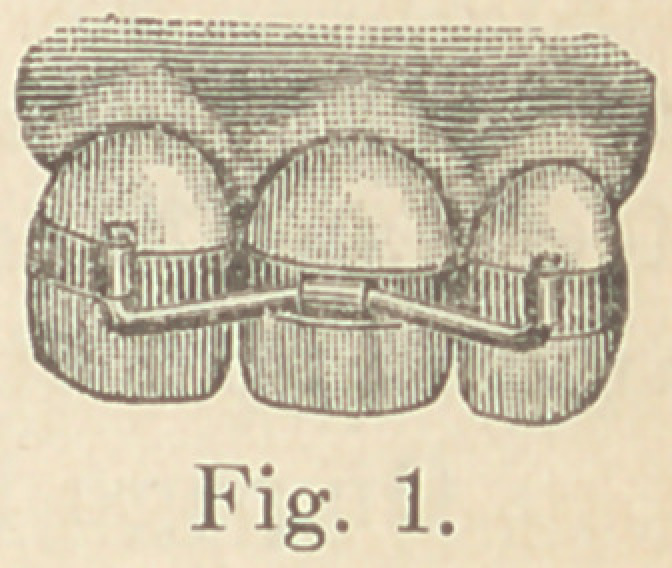# Domestic Correspondence

**Published:** 1889-06

**Authors:** 


### Domestic Correspondence.

To the Editor:

As the planting of teeth, is now becoming so common an operation, and probably destined to become more so, the importance of furnishing some means whereby a planted tooth may be firmly held and supported while it is becoming firm in its new position cannot be over-estimated. Lack of proper support is probably the cause of a large percentage of failures.

Fig. 1 illustrates a very simple and efficient splint for this purpose. It is easily and quickly applied, and may be worn any length of time without inconvenience to the patient. On each side of the space in which the tooth is to be planted is selected a suitable tooth. A strip of band material (shown at F, Fig. 3, page 324) is made into the form of a loop, slipped over the tooth and drawn tightly about the same with a pair of flat-nosed pliers. It is then removed and soldered at the point of union, and the ends clipped off. Delicate pipes (as shown at R, Fig. 3, page 324) are now soldered to each of these bands, on the labial side and parallel to the axis of the tooth. After carefully drying the teeth, the bands are cemented in position. This may be done severa hours or even days in advance of the operation of planting.

Fig. 1.

The tooth to be planted is banded and piped in the same manner already described, only the pipe is soldered to the band at right angles to the tooth. A piece of the gold wire of suitable length is cut (shown at G, Fig. 2, page 324), which exactly fits these pipes. It is slipped through the pipe on the tooth to be planted, and each end is bent at right angles. The tooth is slipped into the socket already prepared, the ends of the wire are slipped through the pipes on the anchor teeth, and, as they pass through, are snipped off with a pair of wire cutters. This will also flatten the ends slightly, which will prevent the splint from coming out of the pipes.

I have frequently dispensed with the band and pipes which encircle the planted tooth, using instead a silk ligature, tying the same tightly around both tooth and splint. This simplifies the operation, and in most instances is quite sufficient.

Dr. John H. Martindale, of this city, has used this form of splint with success in a number of cases for the support of teeth which have been loosened by alveolar necrosis or pyorrhœa.

The advantages of this little device in ease of application, comfort to the patient, and, above all, cleanliness, offering so little refuge for bacteria, will, I think, be readily appreciated by all.

Minneapolis, May 25, 1889. Edward H. Angle.

To the Editor:—Enclosed I forward to you an exact copy of Dr. John Greenwood's advertisement, which I found in the New York City Directory for 1786, taken from the newspaper abstracts of that period (1785), entitled, "Annals of New York City." The originals were published February 28, and November 20, 1786, respectively. The Greenwood referred to below was General Washington's dentist.

A paragraph on page 200, reads thus, "J. Greenwood, dentist, real maker of artificial teeth, makes and sets in teeth so exact as not to be distinguished from the natural, they are not to be taken out at night as some falsely suppose. He likewise transplants natural teeth and fixes them upon gold. He will wait upon ladies and gentlemen at their houses, and may be spoke with at No. 21, John St."

On page 110 is another as follows:

"John Greenwood, dentist, No. 199 Water Street, substitutes artificial teeth in so neat a manner, as not to be perceived from the natural; they give a youthful air to the countenance."

I send these for the Journal if you think it worth while. Note the style of language, which I reproduce exact.

New York City,
May 14, 1889. J. N. Farrar.

---

To the Editor.—Although vigorously opposing every form of implantation of *natural* teeth, the criticism in the May number of *The International Dental Journal*, by Dr. E. A. Bogue, of an implantation operation performed by Dr. R. Ottolengui, and described by him in the January number of the Dental Cosmos, compels me to come to the defense of this method of uniting teeth together.

I have advised this manner of preserving the usefulness of teeth suffering in the last stages of Pyorrhœa Alveolaris before several assemblages, and with the knowledge personally given that many dentists have adopted this operation, it seems fit that I should champion it when attacked in the insidious manner. It is quite evident that Dr. Bogue, who is so keen and intelligent an observer and also an excellent operator, has never seen one of these operations, or he never would have made the contradictory statements soamusing to one who has seen the operation.

The lamented Marshall H. Webb, was often heard to remark that he "preferred one of his gold fillings to the best tooth strucutre ever created."

Dr. Bogue in his criticism supplements this by the following: "If all crevices and crannies existing in a tooth can be filled with any indestructible substance, and the surface made flush with the smooth surface of the tooth, so that it can be kept clean from deposits on every side, that tooth is practically free from decay s long as it is kept clean." Now this is exactly what has been done in Dr. Ottolengui's operation, and is the only way such operations should ever be performed ; that is to leave them, as any good operator would leave his best specimen of a gold filling. The operation was devised to hold teeth, however loose, in a firm position and to *prevent the assemblage of bacteria*, while every other form of holding loose teeth in a firm position, materially assisted in gathering around these teeth immense numbers of the various forms of bacterial life.

This is why Dr. Bogue's criticism seems amusing. To quote further from Dr. Bogue ; " before a wall of perfect enamel, germs are inoperative and powerless. Nature's form and arrangement of perfect teeth, is such that this wall must be breached before these microscopic enemies can get any foothold." In performing this operation the groove is always made through the cutting edges of the teeth a position where this *wall of perfect enamel* is *breached* early in life ; in fact in cases like the one reported in the January *Cosmos* (involving the inferior incisors), the enamel soon disappears entirely from the cutting edges, as it had disappeared in the case of Dr. Andrews. According to Dr. Bogue's own statement which I have already quoted, he admits that such surfaces as these present a most inviting field for the legions of bacterial scavengers to *promote* caries, in the exposed dentinal structures.

I cannot see how the filling of such *crevices* and *crannies* with gold which has been made flush and smooth with the unbroken wall of enamel on every side, can be made a *special nest* for the bleeding of *multifarious bacteria.* MEYER L. RHEIN.

No 104 E. 58th Street, New York.

## Figures and Tables

**Fig. 1. f1:**